# Supplementary material for: An Interaction Network of RNA-Binding Proteins Involved in Drosophila Oogenesis
Source: Mol Cell Proteomics. 2020 Nov 25;19(9):1485–502. doi: 10.1074/mcp.RA119.001912 (PMC8143644; doi:10.1074/mcp.RA119.001912)
Supplement: Supplementary file 1 [file mmc1.zip › mmc1/157750_1_supp_536530_qry2q7.zip!/Single_peptide_identifications_R17-R22/20170411_CO_0685FuBo_R17R18R19_Mix1_16064.pdf]

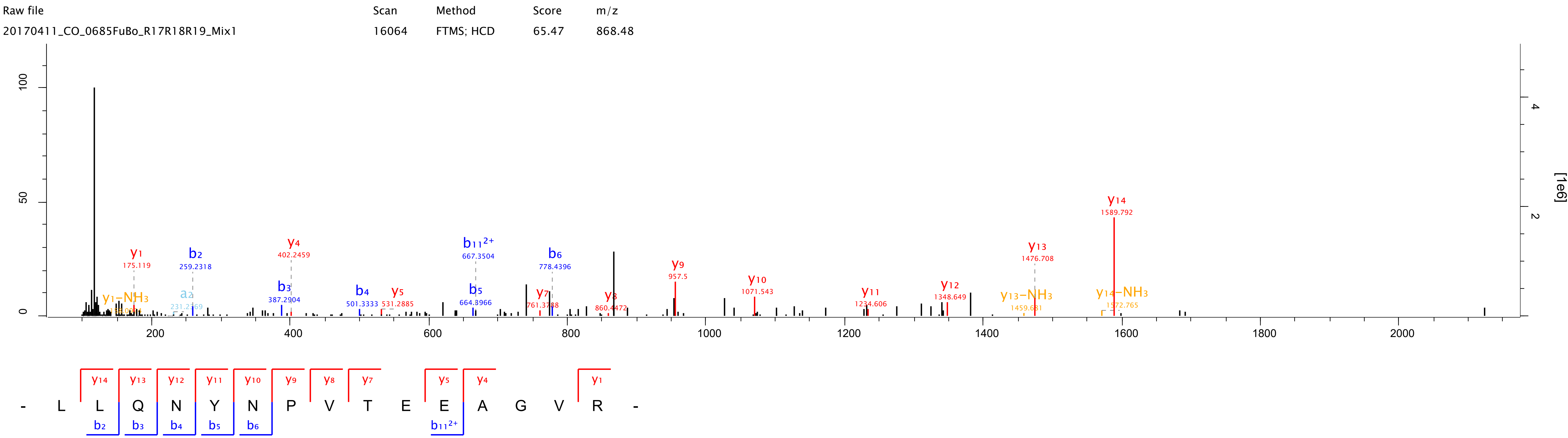
Raw file
20170411_CO_0685FuBo_R17R18R19_Mix1
Scan
16064
Method
FTMS; HCD
Score
65.47
m/z
868.48
y1 175.119
y1-NH3
a2 231.2169
b2 259.2318
b3 387.2904
y4 402.2459
b4 501.3333
y5 531.2885
b11 2+ 667.3504
b5 664.3966
b6 778.4396
y7 761.3788
y8 860.4472
y9 957.5
y10 1071.543
y11 1234.606
y12 1348.649
y13 1476.708
y13-NH3 1459.681
y14 1589.792
y14-NH3 1572.765
[1e6]
- L L Q N Y N P V T E E A G V R -
